# Genetic imaging of the association of oxytocin receptor gene (OXTR) polymorphisms with positive maternal parenting

**DOI:** 10.3389/fnbeh.2014.00021

**Published:** 2014-01-03

**Authors:** Kalina J. Michalska, Jean Decety, Chunyu Liu, Qi Chen, Meghan E. Martz, Suma Jacob, Alison E. Hipwell, Steve S. Lee, Andrea Chronis-Tuscano, Irwin D. Waldman, Benjamin B. Lahey

**Affiliations:** ^1^Department of Psychology, The University of ChicagoChicago, IL, USA; ^2^Section on Development and Affective Neuroscience, National Institute of Mental Health, National Institutes of HealthBethesda, MD, USA; ^3^Department of Human Development, University of MarylandCollege Park, MD, USA; ^4^Department of Psychiatry, The University of ChicagoChicago, IL, USA; ^5^Department of Behavioral Neuroscience, The University of ChicagoChicago, IL, USA; ^6^Department of Psychology, University of MichiganAnn Arbor, MI, USA; ^7^Department of Psychiatry, University of MinnesotaMinneapolis, MN, USA; ^8^Department of Psychiatry, University of PittsburghPittsburgh, PA, USA; ^9^Department of Psychology, University of California Los AngelesLos Angeles, CA, USA; ^10^Department of Psychology, University of MarylandCollege Park, MD, USA; ^11^Department of Psychology, Emory UniversityAtlanta, GA, USA; ^12^Department of Health Studies, The University of ChicagoChicago, IL, USA

**Keywords:** maternal parenting oxytocin receptor gene, functional magnetic resonance imaging

## Abstract

**Background:** Well-validated models of maternal behavior in small-brain mammals posit a central role of oxytocin in parenting, by reducing stress and enhancing the reward value of social interactions with offspring. In contrast, human studies are only beginning to gain insights into how oxytocin modulates maternal behavior and affiliation.

**Methods**: To explore associations between oxytocin receptor genes and maternal parenting behavior in humans, we conducted a genetic imaging study of women selected to exhibit a wide range of observed parenting when their children were 4–6 years old.

**Results**: In response to child stimuli during functional magnetic resonance imaging (fMRI), hemodynamic responses in brain regions that mediate affect, reward, and social behavior were significantly correlated with observed positive parenting. Furthermore, single nucleotide polymorphisms (SNPs) (rs53576 and rs1042778) in the gene encoding the oxytocin receptor were significantly associated with both positive parenting and hemodynamic responses to child stimuli in orbitofrontal cortex (OFC), anterior cingulate cortex (ACC), and hippocampus.

**Conclusions**: These findings contribute to the emerging literature on the role of oxytocin in human social behavior and support the feasibility of tracing biological pathways from genes to neural regions to positive maternal parenting behaviors in humans using genetic imaging methods.

## Introduction

Dysfunctional maternal parenting during early childhood is a robust risk factor for mental and physical disorders associated with mortality in humans (Wegman and Stetler, 2009). In spite of its public health importance, however, the neurobiology of dysfunctional maternal behavior in humans is poorly understood. Research with rodents highlights the involvement of oxytocin (OT) in the initiation and expression of maternal approach behavior, affiliation, and attachment with young (Lim and Young, 2006). Such behaviors correlate with both peripheral measures of OT in plasma and the expression of oxytocin receptors (OXTR) in the brain (Francis et al., 2002). In line with these observations, the OXTR gene single nucleotide polymorphism (SNP), rs53576, in humans, has been associated with a broad range of social behaviors potentially relevant to adaptive parenting behavior (Costa et al., 2006; Israel et al., 2009; Rodrigues et al., 2009). Further elucidating the associations between oxytocinergic polymorphisms and both behavioral and neural phenotypes may provide complementary evidence for the involvement of OT in human parenting. Here we combine functional magnetic resonance imaging (fMRI) and genetic analyses to trace biological pathways from genes to neural circuits to human maternal parenting. We examine whether the same OXTR polymorphisms are associated both with variations in observed maternal parenting and variations in maternal neural responses to child stimuli.

A consensus regarding neurobiological mechanisms of maternal behavior in small-brain mammals has emerged (Brunton and Russell, 2008; Numan and Woodside, 2010). In such animals, being a mature female does not provide a sufficient basis for maternal behavior. Rather, nulliparous females often avoid and even attack newborns (Numan and Insel, 2003). Current models posit that rising levels of estrogens during pregnancy “prime” key brain regions, initiating a process that reverses the valence of infant stimuli from aversive to rewarding, largely by upregulating OT receptors (Carter, 1998; Numan and Woodside, 2010). Much of the knowledge regarding the ability of OT to regulate social interactions is based on data from animals using centrally administered agonists and antagonists or knockout mice. OXTR gene knockout mice are profoundly deficient in maternal behavior (Ragnauth et al., 2005; Takayanagi et al., 2005). In rats, intra-cranial injection of OT following estrogen priming induces maternal behavior in virgin females (Campbell, 2008). Reciprocally, an OXTR antagonist inhibits the natural postpartum onset of maternal behavior (Insel, 2010). Although initial studies suggest similar social effects on humans (see Bartz and Hollander, 2006, for a review), the relevance of these animal findings for humans is not yet clear.

Oxytocin can function as a synaptic neurotransmitter by being released diffusely into extracellular fluid from dendrites, influencing distant neural sites (Landgraf and Neumann, 2004; Ludwig and Leng, 2006; Ross and Young, 2009; van den Burg and Neumann, 2011). Recent morphological and functional data also demonstrate that OT neurons emanating from the hypothalamus have long-range axonal projections capable of releasing OT (Ross et al., 2009; Knobloch et al., 2012). OT directly and indirectly influences sites involved in reward, affect, social cognition, and emotion regulation, particularly the midbrain, striatum, amygdala, hippocampus, anterior cingulate cortex (ACC), and orbitofrontal cortex (OFC) (Olazabal and Young, 2006; Campbell, 2008). It has been hypothesized that the upregulation of OT receptors during late pregnancy and parturition leads directly or indirectly, via dopaminergic reward circuits, to reinforcing interactions with offspring, reduced avoidance of offspring, adaptive maternal behavior, and increased protective maternal aggression against intruders in small-brain mammals (Numan and Woodside, 2010).

Given this literature we focus our attention on a gene with conceptual linkages to caregiving behaviors and parenting. The human OXTR gene is located on chromosome 3p25, containing four exons and three introns. An SNP in the third intron of OXTR, rs53576 (G/A), has been identified as a candidate gene underlying social behavior in humans (Inoue et al., 1994). Only a small literature exists on associations of OXTR gene polymorphisms with human behavior. Individuals with the TT genotype of the rs1042778 SNP in the 3′ untranslated region of OXTR allocated more resources to an unseen opponent in a competitive game (Israel et al., 2009). Individuals with the GG genotype of the intronic SNP rs53576 in OXTR exhibited higher dispositional empathy and less stress reactivity (Rodrigues et al., 2009), whereas the AA genotype was associated with slightly higher depression and lower self-esteem scores (Saphire-Bernstein et al., 2011). In another study, however, the AA genotype of rs53576 was associated with lower positive affect in males only and not related to negative affect or loneliness (Lucht et al., 2009).

Tost et al. (2010) used a partial genetic imaging paradigm to conduct tests of associations between genetic polymorphisms and both neural and behavioral phenotypes. The rs53576 GG genotype was associated with greater dependence on the approval of others and greater amygdala activation and lower amygdala-hypothalamus coupling in functional magnetic imaging (fMRI) in a face-matching task (Tost et al., 2010). The GG genotype was associated with smaller hypothalamic volume, which in turn was correlated with dependence on the approval of others, but predominantly in males. No tests of associations between brain regions activated in fMRI and dependence on social reward were reported, however.

For purposes of the present study, the existing literature has limitations. Most studies used college students (Israel et al., 2009; Rodrigues et al., 2009; Saphire-Bernstein et al., 2011) or volunteers screened to exclude mental disorders (Tost et al., 2010), minimizing both women with children and participants with impaired psychological functioning. Interestingly, the findings of the one study of a representative sample were inconsistent with the studies of less representative samples (Lucht et al., 2009). Additionally, there is limited evidence that the behavioral phenotypes used in these studies are correlated with variations in maternal parenting, except for depressive symptoms and negative affect, which have only been limitedly linked to rs53576 (Saphire-Bernstein et al., 2011).

A few studies of the relevance of OT to human maternal parenting have been published recently, however. One found that plasma OT during pregnancy and the postpartum was positively correlated with adaptive maternal parenting (Levine et al., 2007). In addition, intranasal OT decreased amygdala activation and increased activation of the insula and inferior frontal gyrus when women heard infant cries (Riem et al., 2011a).

Directly relevant to the present study, a study of Caucasian mothers of 2-year old children with behavior problems found that mothers with the OXTR rs53576 GG genotype displayed more sensitive parenting (Bakermans-Kranenburg and van Ijzendoorn, 2008). In a study with more complex findings, however, nulliparous adult females with the rs53576 GG genotype had greater heart rate responses to infant cries, but only among women with low depression scores, with the opposite finding for depressive women (Riem et al., 2011b).

To further lay a foundation for large-scale studies of the neurobiology of dysfunctional maternal parenting, we conducted a study of mothers selected based on their scores on positive and negative parenting of their 4–6 year old children. Using a full genetic imaging paradigm, we tested hypotheses that OXTR gene polymorphisms are associated with variations in both neural responses to child stimuli and maternal parenting, and that neural responses in the same regions are correlated with maternal parenting. fMRI studies support such genetic imaging studies of human maternal parenting by showing that infant faces and cries activate maternal brain regions shown to contain OT receptors in rodents (i.e., amygdala, hippocampus, and striatum) (Lee et al., 2009; Stoop, 2012), and midbrain and basal ganglia regions involved in reward (Lorberbaum et al., 2002; Bartels and Zeki, 2004; Swain et al., 2007). In more experienced mothers of older children, amygdala and insula activations are not typically observed, however, but regions involved in social cognition, such as the OFC, medial prefrontal cortex (MPFC), and superior temporal sulcus (STS), are activated (Leibenluft et al., 2004; Swain et al., 2004; Swain, 2011). No fMRI studies have examined associations between OXTR gene variants and variations in both maternal parenting and maternal neural responses to child stimuli in a genetic imaging paradigm.

## Methods

### Participants

Seventeen female and 104 males 4–6 year olds were recruited from a child psychiatry clinic in Chicago during 1994–1995 for a longitudinal study of children with attention-deficit/hyperactivity disorder (ADHD) and matched controls (Lahey et al., 2005; Chronis-Tuscano et al., 2010; Lee et al., 2010). Participants taking short-acting stimulant medications were included, but all assessments were conducted when the child was off medication. Previous reports from this study revealed that the measures of positive and negative maternal parenting used predicted poor outcomes of children with ADHD through adolescence, controlling for multiple factors (Chronis et al., 2007; Lahey et al., 2011). Thus, the genetic and neural correlates of measures of maternal parenting already known to predict long-term child outcomes can be studied in this sample. In 2009–2010, 40 mothers were selected for the present study by recruiting from the extremes of positive and negative parenting scores to maximize variation in parenting. Participants' written consent was obtained. All participants were paid for their participation. The study was approved by the University of Chicago Institutional Review Board and conducted in accordance with the Declaration of Helsinki. All of the observed children had written informed consent from their biological mothers, all of whom were custodial. Characteristics of participants are in Supplemental Table S1.

### Mother-child interaction task

Mother-child interactions were videotaped in a room equipped with furniture, toys and other objects, and a television showing cartoons (Chronis et al., 2007). Mothers were invited to play freely with their children. After 10 min, the interviewer returned and scattered clothes, papers, and empty containers around the room. The mother was given an Etch-a-Sketch, worksheets, a magazine, a pencil, and written instructions to complete tasks with her child in order over 15 min: (1) return toys to shelves, (2) put clothes in the box, (3) put paper and containers in the wastebasket, (4) count geometric shapes, (5) copy a set of geometric designs on paper, (6) dust the table with a cloth, (7) draw a diagonal line on the Etch-a-Sketch, and (8) choose one toy and play quietly while the mother reads and takes a telephone call. The first 13 min of the task situation were coded because some dyads completed all tasks early.

Interactions were coded using the Dyadic Parent-Child Interaction Coding System (DPICS) (Robinson and Eyberg, 1981). Two reliable and valid measures of parenting were used by averaging standardized scores across the structured and play situations: Positive parenting (praise, positive affect, and physical positive) and negative parenting (negative commands, critical statements, and physical negatives) (Robinson and Eyberg, 1981; Webster-Stratton, 1998). These measures predict long-term child outcomes in this sample (Chronis et al., 2007; Lahey et al., 2011). Thirty percent of the videotapes were coded by a reliability coder. Inter-observer agreement was 0.95 for positive parenting, 0.90 for negative parenting, and 0.92 for total child disruptive behavior.

### Neuroimaging

Photographs of the child at 4–6 years from the videotapes and photos of unrelated demographically matched children were used in fMRI as stimuli. Mothers also viewed a series of dynamic visual stimuli, each consisting of three 600 × 480 pixel color photographs presented successively for 1000, 200, and 1000 ms, respectively to imply motion. Forty-eight dynamic stimuli portrayed misbehaviors (e.g., a child intentionally kicking a female adult on the leg), and 48 portrayed parallel neutral behaviors (e.g., a child standing next to a female adult) without showing faces. The stimuli were matched on numbers of people and objects and varied in skin color.

Stimuli were presented with E-prime 1.2 (PsychologySoftware Tools, 2011) by back-projection in a block design. Dynamic child stimuli were presented in 16 19.8 s blocks and a fixation cross was presented in 16 18 s baseline blocks. Stimuli were blocked by type (provocative/neutral child behavior), each consisting of 6 stimuli (2200 ms each) with six 1100 ms inter-stimulus intervals, during which a black fixation cross was presented against a gray background. Participants were shown the stimuli in 2 sessions (8 active blocks per session). In one session, immediately preceding every stimulus block, mothers were shown a photograph of their own child for 6 s and instructed to “imagine this is your child” in the actions that followed. In the other session, mothers were shown a photograph of a demographically matched child and instructed to “imagine this is not your child” in the actions that followed. Participants were instructed to watch the stimuli carefully and no overt response was required. Session order was counterbalanced across participants.

MRI was performed on a 3-T Philips Achieva Quasar scanner. The fMRI pulse sequence parameters include time repetition/time echo (TR/TE) 2000/25, flip angle = 77, 32 contiguous slices with 4 mm thickness, slice gap 0.5 mm, 224 × 224 mm^2^ field of view (FOV), approximately 64 × 64 matrix. High resolution structural images were acquired in the sagittal plane using a T1-weighted 3D Turbo Field Echo (TFE/MP-RAGE) anatomical scan with the following parameters: *TR* = 8.1 ms, *TE* = 3.7 ms, *FOV* = 224 × 224 × 160 mm^3^, isotropic voxel size 1 × 1 × 1 mm^3^, matrix size 224 × 224. During anatomical scans after the stimulation paradigms, participants watched a movie about tropical beaches.

### Genotyping

DNA from mothers was isolated from saliva in Oragene kits (DNAGenotek, 2010) and checked for quality by OD ratio of 260/280 and concentration. Genotyping rs53576 and rs1042778 was performed using TaqMan pre-designed SNP genotyping assays (AppliedBiosystems, 2010). PCR was carried out in a total volume of 3 μ l containing 10 ng genomic DNA, 1.5 μ l 2×TaqMan universal PCR master mix (AppliedBiosystems, 2010), 0.075 μ l 40× SNP genotyping assay. After 95°C 10 min, 40 cycles consisting of 15 s at 92°C and 1 min at 60°C annealing temperature were performed. After PCR amplification, an endpoint plate read using a 7900 Real-Time PCR System was performed. The Sequence Detection System (AppliedBiosystems, 2010) was used to call genotypes.

### Statistical analyses

#### fMRI analysis

Image processing was carried out with SPM8 in MATLAB 7.0 (Marsh et al., 2008). Preprocessing included correction for head motion, normalization to the SPM8 echo-planar imaging template, and smoothing using a 6-mm full-width half-maximum isotropic Gaussian kernel. Images were realigned and normalized using standard SPM procedures. All participants had less than 0.5 voxels of in-plane motion throughout scanning. A 2-level approach for block-design fMRI data was adopted using SPM8. A voxel-by-voxel multiple regression analysis of expected signal changes for the child photographs and the 2 block categories, constructed using the SPM8 hemodynamic response function, was applied to preprocessed images. Individual subject data were analyzed using a fixed-effects model. Group data were analyzed using a random-effects model. Condition effects at the subject level were modeled by box-car regressors representing type of child photograph and the two block types.

The fMRI contrasts that were examined across participants were: (1) Own vs. other child image preceding each of the action blocks and (2) provocative vs. neutral child behavior in the own-child session. Activations were overlaid on a representative high-resolution structural T1-weighted image from one subject from the SPM8 canonical image set, coregistered to Montreal Neurological Institute (MNI) space.

To additionally test for activations previously reported in maternal parenting studies, we selected five regions of interest (ROIs) identified in previous published work on the neuroscience of maternal parenting (Swain, 2011) and about which we had *a priori* hypotheses, using a small volume correction (SVC) for family wise error (FWE) at *P* < 0.05. The following bilateral regions were selected: amygdala, hippocampus, anterior insula, ACC, and OFC. Analyses were implemented in SPM8 based on corrections for multiple comparisons confined to an ROI (Worsley et al., 1996). Data extraction for 5-mm spherical ROIs was performed using the rfxplot toolbox (Glascher, 2009) in SPM8. Coordinates were based on results from the whole-brain analyses and neuroanatomical atlases. The small volumes consisted of a 5 mm sphere centered at the most significant voxel of the clusters activated at *P* < 0.001 uncorrected in the whole brain analysis.

#### Tests of association in genetic imaging paradigm

Three sequenced and prioritized sets of generalized linear models (Nelder and Wedderburn, 1972) were conducted as illustrated in Supplemental Figure S1:

1. Additive terms were coded −1, 0, and 1 to jointly test linear differences among 0, 1, or 2 minor A alleles for rs53576 and G alleles for rs1042778. Non-additive terms, coded −1, 2, and −1, jointly captured any mode of non-additive transmission for each SNP. Additive and non-additive terms were run in the same model. In two separate analyses, positive and negative maternal parenting were regressed on the four OXTR terms (additive and non-additive terms for rs53576 and rs1042778), simultaneously controlling child's sex, race-ethnicity, birth order, age in wave 1, mother's age at scanning, delivery (Caesarian or vaginal), and total parity. To adjust for child effects on the mother's parenting (Bell and Chapman, 1986), the child's diagnosis of ADHD and disruptive behavior during the mother-child interaction also were simultaneous covariates (Lahey et al., 2011).
2. For each parenting measure for which the tests of genetic associations were significant at *p* < 0.05 in step 1, generalized linear models regressed parenting on each of the five bilateral *a priori* selected ROIs, with the same covariates plus time of fMRI scanning.
3. For each of the five *a priori* selected ROIs that was significantly associated with parenting in step 2, regression models with the same covariates as step 2 tested associations between the SNP and the ROI.

## Results

### Whole-brain analyses

**Figure 1:**
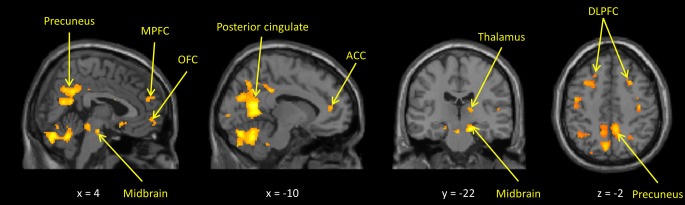
**Maternal hemodynamic brain activations in response to photographs of her own vs. an unrelated child (*p* < 0.005, uncorrected)**. See Supplemental Table S2 for corrected activations.

**Figure 2:**
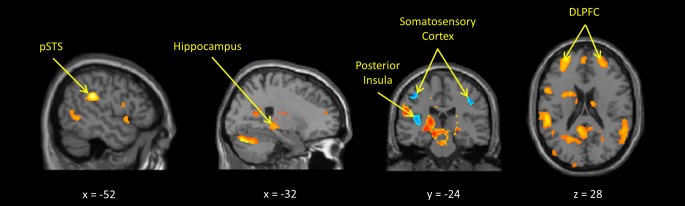
**Maternal hemodynamic brain activations in response to dynamic images of a young person behaving inappropriately vs. appropriately (*p* < 0.005, uncorrected)**. See Supplemental Table S3 for corrected activations.

In the whole-brain analyses, the own > other child contrast was associated with hemodynamic increases in regions subserving motivation, reward, and emotion regulation processing (including the midbrain, dorsal putamen, thalamus, anterior cingulate, and prefrontal cortices) and previously been found to be activated by child stimuli (Swain, 2011). The inappropriate > neutral behavior contrast activated similar regions, with additional activations in areas involved in social cognition such as the posterior STS and deactivations in somatosensory cortex and bilateral posterior insula (Figures 1, 2 and Supplemental Tables S2, S3).

### Associations between SNPs and parenting

Allele frequencies are shown in Supplemental Table S4. There were no significant ancestry group differences in allele frequencies for rs53576, but the T allele of rs1042778 was more common in African Americans, requiring ancestry to be covaried. There were no significant associations between OXTR SNPs and negative parenting, but rs53576 and rs1042778 each accounted for unique variance in positive maternal parenting, although only the additive association between rs53576 alleles and positive parenting was significant at *p* < 0.05/8 = 0.006 corrected for multiple testing (Table 1). A subsequent regression analysis revealed significant interactions between ancestry and rs53576 (non-additive term: χ^2^ = 6.05, *P* < 0.02) and rs1042778 (additive term: χ^2^ = 10.11, *P* < 0.002). Although cell sizes were too small to interpret these interactions, they raise the possibility that OXTR polymorphisms may be associated with parenting differently in mothers of African and European descent.

**Table 1:** **Simultaneous associations of each term for additive and non-additive associations (each 1 DF) between OXTR SNPs with positive and negative maternal parenting (in separate analyses), adjusting for all covariates; *N* = 35**.

	**Wald**	
	**χ^2^**	***P =***
**POSITIVE PARENTING**		
rs53576 (additive)	15.43	<0.0001
rs53576 (non-additive)	0.02	0.8814
rs1042778 (additive)	2.93	0.0872
rs1042778 (non-additive)	5.37	0.0205
**NEGATIVE PARENTING**		
rs53576 (additive)	0.09	0.7647
rs53576 (non-additive)	0.01	0.9339
rs1042778 (additive)	1.00	0.3178
rs1042778 (non-additive)	0.01	0.9305

*Simultaneous covariates: Child's age in years, child's sex, African American ancestry, child's ADHD diagnosis, child's disruptive behavior during mother-child interaction task, first born status, mother's number of live births, prematurity in weeks, Caesarian delivery, and mother's age at scanning*.

### Associations between ROIs and parenting

In separate analyses for each ROI, we observed associations between observed positive parenting and activation in bilateral OFC and left ACC when mothers viewed their own vs. another child, at *P* = 0.05 levels (see Table 2). Mothers who scored higher on observed positive parenting behaviors showed an increase in hemodynamic response in OFC and ACC when they viewed pictures of their own child, compared to pictures of another child.

**Table 2:** **Tests of associations between observed maternal positive parenting and maternal BOLD activations by the two kids of stimuli in brain regions of interest using small-volume correction (*N* = 34)**.

**Cortical regions of interest**	**Positive Parenting**	
	**β**	**χ^2^**
**OWN > UNRELATED CHILD**		
**Orbital frontal cortex**		
Left	**0.25**	4.56^*^
Right	**0.26**	4.60^*^
**Anterior cingulate cortex**		
Left	**0.43**	5.64^*^
Right	0.12	0.44
**PROVOCATIVE > NEUTRAL BEHAVIOR WHEN VIEWING OWN CHILD CONTRAST**		
**Anterior cingulate cortex**		
Left	−0.15	0.10
Right	**−0.97**	5.87^*^
**Hippocampus**		
Left	0.18	0.08
Right	**1.42**	3.85^*^

* *P < 0.05. Simultaneous regression analyses controlling for the child's sex and age at the time of the mother-child interaction task, the child's race-ethnicity, the child's diagnosis (ADHD or healthy control), the child's level of disruptive behavior during the task, premature birth, caesarian delivery, parity, birth order, time of day of the mother's scan, and mother's age at the time of the scan*.

Two associations between ROIs activated in the inappropriate > appropriate child behavior contrast and positive parenting were significant at *P* = 0.05 levels. Associations were observed in the right ACC and right hippocampus (see Table 2). Because we focused our analyses on specific regions, hypothesized *a priori*, ROIs above a *P* = 0.05 uncorrected threshold were included in the next step. It should be noted, however, that none of the analyses were significant at α = 0.05/20 = 0.0025 corrected for multiple testing.

### Associations between SNPs and ROIs

**Figure 3:**
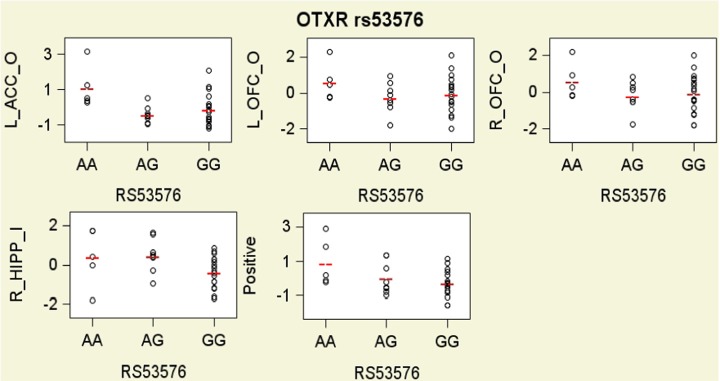
**Scatter plots with group means residualized on all covariates for hemodynamic responses in the own/other child (O) contrast and the inappropriate/appropriate behavior contrast (I) in brain regions of interest that were found to be significantly related to oxytocin and vasopressin genotypes, and for observed maternal parenting, presented by oxytocin receptor gene (OXTR) rs53576 genotypes**. To facilitate interpretation, all variables on y axes are expressed as sample z-scores (mean = 0; standard deviation = 1).

As shown in Figure 3 and Table 3 tests of additive associations between rs53576 and the ROIs that were both significantly activated by child stimuli and were significantly associated with positive parenting, were significant at *p* < 0.05 uncorrected levels. Specifically, these were in bilateral OFC, left ACC and right hippocampus. Further, the association between left ACC activation and rs53576 (additive) in the own > other child contrast and between right hippocampus activation and rs53576 (additive) in the inappropriate > appropriate behavior contrast remained significant after Bonferroni correction (α = 0.05/20 = 0.0025).

**Table 3:** **Results of simultaneous regression analyses testing associations of each additive and non-additive genetic term (each *DF* = 1) for OXTR rs53576 and rs1042778 with each neural region of interest that was both significantly activated in the fMRI paradigm and found to be significantly associated with observed positive maternal parenting with covariates, conducted separately for each neural region of interest (*N* = 30)**.

	**Wald χ^**2**^**	***P*** <
**OWN CHILD > OTHER CHILD fMRI PROBE PARADIGM**		
*Left orbital frontal cortex*		
** rs53576 (additive)**	**3.90**	**0.0482**
rs1042778 (non-additive)	0.06	0.8080
*Right orbital frontal cortex*		
** rs53576 (additive)**	**4.67**	**0.0306**
rs1042778 (non-additive)	0.00	0.9806
*Left anterior cingulate cortex*		
** rs53576 (additive)**	**29.07**	**<0.0001**
rs1042778 (non-additive)	0.38	0.5398
**INAPPROPRIATE > NEUTRAL CHILD BEHAVIOR fMRI PROBE PARADIGM**		
*Right anterior cingulate cortex*		
rs53576 (additive)	0.10	0.7552
rs1042778 (non-additive)	0.26	0.6113
*Right hippocampus*		
** rs53576 (additive)**	**17.42**	**<0.0001**
** rs1042778 (non-additive)**	**5.69**	**0.0171**

*Simultaneous covariates in each model: child's age in years, child's sex, African American ancestry, child's ADHD diagnosis, child's disruptive behavior during mother-child interaction task, first born status, mother's number of live births, prematurity in weeks, Caesarian delivery, time of day of scanning, and mother's age at scanning*.

In addition, one test of independent associations between rs1042778 (non-additive) and these ROIs were significant at *p* < 0.05 levels (uncorrected). This was in the right hippocampus. None were significant at α = 0.05/20 = 0.0025 levels.

## Discussion

Consistent with previous studies of human mothers (Lorberbaum et al., 2002; Nitschke et al., 2004; Swain et al., 2007, 2008), two kinds of child stimuli activated neural regions implicated in face processing, reward processing, social cognition, and executive control of emotion (Figures 1, 2 and Supplemental Tables S2, S3). More importantly, *individual differences* in maternal neural responses in key ROIs in both paradigms were associated with variations in maternal parenting.

Notably, the present findings are *internally consistent*. Not only was the rs53576 A allele associated with higher levels of positive parenting, it also was associated with greater activation in the own > other child contrast in two a priori hypothesized ROIs that were associated with positive parenting (Figure 3). In particular, the rs53576 A allele was associated with greater activation in ROIs involved in (1) orienting toward, monitoring, and evaluating child cues (Parsons et al., 2013) and other emotional stimuli (bilateral OFC) (Rolls, 2000; Nitschke et al., 2004; Volkow et al., 2011) and (2) assessing the salience of emotional information and regulating emotional responses (left ACC) (Bush et al., 2000; Kerns et al., 2004). The present findings are consistent with previous findings that OFC activation is correlated with positive mood related to maternal behavior (Nitschke et al., 2004). Furthermore, when mothers viewed inappropriate behavior when instructed to imagine it was their own child, the rs53576 A allele was associated with greater activation of the hippocampus. This is interesting given that activation of OT receptors in the hippocampus in rats inhibits behavioral responses to stressors (Cohen et al., 2010). Because child misbehavior often elicits harsh maternal behavior (Lahey et al., 1984, 2011), this may reflect greater inhibition of such responses in more positive mothers.

Mothers with T alleles of OXTR rs1042778 exhibited both greater positive parenting and greater activation of the right hippocampus in the own > other child contrast, but otherwise there was less clarity and internal consistency in the findings for rs1042778. Furthermore, the nature of the non-additive associations of rs1042778 with the hippocampal ROI, and its relation to positive parenting, is unclear.

Nonetheless, it is essential to note that the present study replicated the association between genotype and positive parenting found in the only previous study of rs53576 and parenting (Bakermans-Kranenburg and van Ijzendoorn, 2008) *in reverse*. In the previous study, mothers with GG genotype of rs53576 displayed more sensitive parenting with 2 year olds than mothers with the AA or AG genotype. Because such “allele flip” replications are commonly reported in the genetics literature, Clarke and Cardon (2010) delineated the conditions under which the results of *both* studies could be valid. This is possible when effect sizes are large, the samples differ in ancestry composition, and the target polymorphism interacts with unmeasured polymorphisms that vary in allele frequencies among ancestry groups (Clarke and Cardon, 2010). Because the first two conditions are met in this case, interactions of both SNPs with other polymorphisms that differ in allele frequency in persons of African and European descent could result in the differences in findings for rs53576 in the two studies. Thus, the present findings could be a valid “allele flip” replication of the previous studies (Bakermans-Kranenburg and van Ijzendoorn, 2008). No conclusions can be reached, of course, until the hypothesis that the direction of associations between genotypes of rs53576 and parenting differ by ancestry group are tested in the future.

One potential difference between these two studies is that mothers in the present study were rated on *positive* parenting behaviors, whereas the study by Bakermans-Kranenburg and van Ijzendoorn assessed *sensitive* parenting, which may be separable constructs. A consistently positive mother, for instance, may not always be sensitive to her child's needs, while a sensitive mother may not always engage in positive parenting behaviors.

It is important to understand the present findings in the context of several issues and limitations:

### Limited markers

Only two SNPs were used to characterize variation in the OXTR gene. Thus, although the present findings support further research, future studies should genotype all polymorphisms in this and related genes to fully understand their impact on neural responses to child stimuli and variations in maternal parenting. This would likely require a larger sample size.

### Conservative statistical strategy

The statistical strategy illustrated in Supplemental Figure S1 limited the statistical analyses of hemodynamic responses to child stimuli to only those regions that were *both* significantly activated by child stimuli and were significantly associated with positive parenting, and only included SNPs that were found to be significantly associated with positive parenting. This was done to focus on the feasibility of testing hypotheses regarding neural pathways from variations in these SNPs to variations in maternal parenting using fMRI-based genetic imaging methods. Thus, for example, tests were not conducted to determine if OXTR gene polymorphisms were associated with hemodynamic responses to child stimuli in ROIs that were not significantly associated with maternal parenting. Thus, these analyses could underestimate the number of ROIs whose functioning is associated with these SNPs.

### Sample size

In principle, the size of a sample is unrelated to the likelihood of rejecting the null hypothesis when there is no association in the population (type I error). This is because the alpha level (e.g., *p* < 0.05) for each individual statistical test is set in advance and the statistical test is automatically penalized for smaller sample sizes. Therefore, the probability that the null hypothesis was rejected incorrectly is not a major issue in the present study. Nonetheless, smaller samples impose three serious limits on statistical testing. First, because the statistical tests used in this study are based on estimations, a degree of error can be expected in smaller samples. Second, the probability of failing to reject the null hypothesis when there is an association in the population (type II error) is jointly related to the sample size and the magnitude of the effect size. Only associations with large effect sizes can be detected in small samples. Therefore, it is possible that the present study failed to detect associations of smaller but still important effect sizes. Third, confidence intervals around effect sizes are larger in smaller samples. This makes it more difficult to determine the sample size needed in future studies. This is because power analyses must be based on the lower limit of the confidence interval for the effect size and smaller sample sizes provide lower estimates of this value.

### Sample representativeness and heterogeneity

A potentially more serious limitation is that the present sample is not representative, partly because smaller samples are inherently less likely to represent the population. In addition, this sample was selected from a single clinic rather than from a representative sampling frame. This means that estimates of the magnitude of associations are unlikely to accurately mirror the population. Furthermore, the present sample also is heterogeneous in terms of race-ethnicity and in the different methods used to sample cases between ADHD and controls. Although a range of demographic variables were controlled in the present analyses, it is possible that the findings are influenced to an unknown degree by artifacts of population stratification of alleles. Future studies must address these issues by using samples that are either more homogeneous or more representative and large enough to adjust for population stratification.

### Time between observation of mother-child interaction and scanning

In this initial study, we used an available sample of mothers on whom mother-child interactions had been observed when the children were 4–6 years old. This allowed us to select the sample for scanning and genotyping by oversampling on the extremes of maternal parenting in a way that ensured a broad distribution of maternal parenting scores to increase statistical power for detecting associations with parenting. For this particular sample, this necessitated scanning the mothers as they viewed stimuli approximately 15 years later. The implications of this time frame must be considered separately for each set of statistical tests.

The 15-year gap has no implications for the tests of association between genotypes and hemodynamic responses of ROIs, as genotyping and scanning were conducted contemporaneously. The gap similarly is not likely to be an issue for the tests of associations between genotypes and parenting as DNA sequences do not change over time. Nonetheless, it is possible that our tests of association between parenting and genotypes are conservative. This is because epigenetic changes that affected expression of those genotypes could have occurred during the intervening 15 years. The gap between the mother-child observations and scanning could have weakened or even distorted these tests of associations between ROI responses and parenting. For instance, the difference in ROI activation between genotypes may potentially reflect an effect of genotype on how mothers remember their child or their child's behavior, or even the status of the current relationship with the child. Relatedly, given this 15-year gap, the mother's current relationship with her child might impact any correlations between positive parenting and hemodynamic activity in ROIs, particularly as the parenting measures were previously found to predict long-term outcomes for this sample (Chronis et al., 2007; Lahey et al., 2011). Nonetheless, the time gap had the effect of focusing the present study on only highly enduring aspects of neurobiological functioning that are related to variations in early maternal parenting. It is also plausible that the perceived effects of genotype on behavior and/or brain response might be due to the presence of the same or similar genotype in either the previous generation (possibly affecting the mother's own upbringing) or in the child (possibly affecting the behavior of the child and, therefore, the associations and responses the mother has toward them).

The ultimate goal of this line of research is to trace environmentally moderated neural pathways from variants in genes to dysfunctional maternal parenting to improve prevention. Such research is essential to understanding why some mothers parent adaptively whereas others are neglectful or abusive. As such, this study was designed to provide a foundation for future research, rather than to conduct strong tests of hypotheses based on previous studies.

To our knowledge, the present study is the first to provide linked evidence on such putative pathways from polymorphisms in OXTR to neural functioning to parenting. These findings support future tests of the hypotheses that variations in genes coding OXTR are related to variations in human maternal parenting. Although the present findings are internally consistent, they are not consistent with a previous study and there are limitations to this study regarding the sample and the time between the observed mother-child interactions and scanning.

### Conflict of interest statement

The authors declare that the research was conducted in the absence of any commercial or financial relationships that could be construed as a potential conflict of interest.

## Supplementary material

Supplemental Figure S1 **Flowchart of the sequenced statistical analyses**.

Supplemental Table S1 **Demographic and birth characteristics of three subgroups of a total 40 mothers and their children participating in tests of associations between different variables**.

Supplemental Table S2 **Areas of significant hemodynamic activation when viewing a picture of own child > unrelated child**.

Supplemental Table S3 **Areas of significant activation and deactivation when viewing a person behaving inappropriately > behaving neutrally in the own child condition**.

Supplemental Table S4 **Allele frequencies of OXTR SNPs by ancestry groups**.

## References

[B2] Bakermans-Kranenburg M. J. van Ijzendoorn M. H. (2008). Oxytocin receptor (OXTR) and serotonin transporter (5-HTT) genes associated with observed parenting. Soc. Cogn. Affect. Neurosci. 3, 128–134 10.1093/scan/nsn004 19015103 PMC2555463

[B3] Bartels A. Zeki S. (2004). The neural correlates of maternal and romantic love. Neuroimage 21, 1155–1166 10.1016/j.neuroimage.2003.11.003 15006682

[B4] Bartz J. A. Hollander E. (2006). The neuroscience of affiliation: forging links between basic and clinical research on neuropeptides and social behavior. Horm. Behav. 50, 518–528 10.1016/j.yhbeh.2006.06.018 16884725

[B5] Bell R. Q. Chapman M. (1986). Child effects in studies using experimental or brief longitudinal approaches to socialization. Dev. Psychol. 22, 595–603 10.1037/0012-1649.22.5.595

[B6] Brunton P. J. Russell J. A. (2008). The expectant brain: adapting for motherhood. Nat. Rev. Neurosci. 1, 11–25 10.1038/nrn2280 18073776

[B7] Bush G. Luu P. Posner M. I. (2000). Cognitive and emotional influences in anterior cingulate cortex. Trends Cogn. Sci. 4, 215–222 10.1016/S1364-6613(00)01483-2 10827444

[B8] Campbell A. (2008). Attachment, aggression and affiliation: the role of oxytocin in female social behavior. Biol. Psychol. 77, 1–10 10.1016/j.biopsycho.2007.09.001 17931766

[B9] Carter C. S. (1998). Neuroendocrine perspectives on social attachment and love. Psychoneuroendocrinology 23, 779–818 10.1016/S0306-4530(98)00055-9 9924738

[B10] Chronis A. M. Lahey B. B. Pelham W. E. Williams S. H. Baumann B. L. Kipp H. (2007). Maternal depression and early positive parenting predict future conduct problems in young children with attention-deficit/hyperactivity disorder. Dev. Psychol. 43, 70–82 10.1037/0012-1649.43.1.70 17201509

[B11] Chronis-Tuscano A. Molina B. S. G. Pelham W. E. Applegate B. Dahlke A. Overmyer M. (2010). Very early predictors of adolescent depression and suicide attempts in children with attention-deficit/hyperactivity disorder. Arch. Gen. Psychiatry 67, 1044–1051 10.1001/archgenpsychiatry.2010.127 20921120 PMC3382065

[B12] Clarke G. M. Cardon L. R. (2010). Aspects of observing and claiming allele flips in association studies. Genet. Epidemiol. 34, 266–274 10.1002/gepi.20458 20013941

[B13] Cohen H. Kaplan Z. Kozlovsky N. Gidron Y. Matar M. A. Zohar J. (2010). Hippocampal microinfusion of oxytocin attenuates the behavioural response to stress by means of dynamic interplay with the glucocorticoid-catecholamine responses. J. Neuroendocrinol. 22, 889–904 10.1111/j.1365-2826.2010.02003.x 20403087

[B13a] Costa B. Pini S. Gabelloni P. Abelli M. Lari L. Cardini A. (2009). Oxytocin receptor polymorphisms and adult attachment style in patients with depression. Psychoneuroendocrinology, 34, 1506–1514 10.1016/j.psyneuen.2009.05.006 19515497

[B14] DNAGenotek. (2010). Oragene DNA Self-Collection Kit. Ottawa, ON: DNAGenotek, Inc

[B15] Francis D. D. Young L. J. Meaney M. J. Insel T. R. (2002). Naturally occurring differences in maternal care are associated with the expression of oxytocin and vasopressin (V1a) receptors: gender differences. J. Endocrinol. 14, 349–353 10.1046/j.0007-1331.2002.00776.x 12000539

[B16] Glascher J. (2009). Visualization of group inference data in functional neuroimaging. Neuroinformatics 7, 73–82 10.1007/s12021-008-9042-x 19140033

[B17a] Inoue T. Kimura T. Azuma C. Inazawa J. Takemura M. Kikuchi T. (1994). Structural organization of the human oxytocin receptor gene. J. Biol. Chem. 269, 32451–32456 7798245

[B17] Insel T. R. (2010). The challenge of translation in social neuroscience: a review of oxytocin, vasopressin, and affiliative behavior. Neuron 65, 768–779 10.1016/j.neuron.2010.03.005 20346754 PMC2847497

[B18] Israel S. Lerer E. Shalev I. Uzefovsky F. Riebold M. Laiba E. (2009). The oxytocin receptor (OXTR) contributes to prosocial fund allocations in the dictator game and the social value orientations task. PLoS ONE 4:e5535 10.1371/journal.pone.0005535 19461999 PMC2680041

[B19] Kerns J. G. Cohen J. D. MacDonald A. W. Cho R. Y. Stenger V. A. Carter C. S. (2004). Anterior cingulate conflict monitoring and adjustments in control. Science 303, 1023–1026 10.1126/science.1089910 14963333

[B20] Knobloch H. S. Charlet A. Hoffman L. C. Eliava M. Khrulev S. Cetin A. H. (2012). Evoked axonal oxytocin release in the central amygdala attenuates fear response. Neuron 73, 553–566 10.1016/j.neuron.2011.11.030 22325206

[B21] Lahey B. B. Conger R. D. Atkeson B. M. Treiber F. A. (1984). Parenting behavior and emotional status of physically abusive mothers. J. Consult. Clin. Psychol. 52, 1062–1071 10.1037/0022-006X.52.6.1062 6520276

[B22] Lahey B. B. Pelham W. E. Loney J. Lee S. S. Willcutt E. (2005). Instability of the DSM-IV subtypes of ADHD from preschool through elementary school. Arch. Gen. Psychiatry 62, 896–902 10.1001/archpsyc.62.8.896 16061767

[B23] Lahey B. B. Rathouz P. J. Lee S. S. Chronis-Tuscano A. Pelham W. E. Waldman I. D. (2011). Interactions between early parenting and a polymorphism of the child's dopamine transporter gene in predicting future child conduct disorder symptoms. J. Abnorm. Psychol. 120, 33–45 10.1037/a0021133 21171728 PMC3058552

[B24] Landgraf R. Neumann I. D. (2004). Vasopressin and oxytocin release within the brain: a dynamic concept of multiple and variable modes of neuropeptide communication. Front. Neuroendocrinol. 25, 150–176 10.1016/j.yfrne.2004.05.001 15589267

[B25] Lee H. J. Macbeth A. H. Pagani J. H. (2009). Oxytocin: the great facilitator of life. Prog. Neurobiol. 88, 127–151 10.1016/j.pneurobio.2009.04.001 19482229 PMC2689929

[B26] Lee S. S. Chronis-Tuscano A. Keenan K. Pelham W. E. Loney J. Van Hulle C. A. (2010). Association of maternal dopamine transporter genotype with negative parenting: evidence for gene x environment interaction with child disruptive behavior. Mol. Psychiatry 15, 548–558 10.1038/mp.2008.102 18779819

[B27] Leibenluft E. Gobbini M. I. Harrison T. Haxby J. V. (2004). Mothers' neural activation in response to pictures of their children and other children. Biol. Psychiatry 56, 225–232 10.1016/j.biopsych.2004.05.017 15312809

[B29] Levine A. Zagoory-Sharon O. Feldman R. Weller A. (2007). Oxytocin during pregnancy and early postpartum: individual patterns and maternal-fetal attachment. Peptides 28, 1162–1169 10.1016/j.peptides.2007.04.016 17513013

[B29a] Lim M. M. Young L. J. (2006). Neuropeptidergic regulation of affiliative behavior and social bonding in animals. Horm. Behav. 50, 506–517 10.1016/j.yhbeh.2006.06.028 16890230

[B30] Lorberbaum J. P. Newman J. D. Horwitz A. R. Dubno J. R. Lydiard R. B. Hamner M. B. (2002). A potential role for thalamocingulate circuitry in human maternal behavior. Biol. Psychiatry 51, 431–445 10.1016/S0006-3223(01)01284-7 11922877

[B31] Lucht M. J. Barnow S. Sonnenfeld C. Rosenberger A. Grabe H. J. Schroeder W. (2009). Associations between the oxytocin receptor gene (OXTR) and affect, loneliness and intelligence in normal subjects. Prog. Neuropsychopharmacol. Biol. Psychiatry 33, 860–866 10.1016/j.pnpbp.2009.04.004 19376182

[B32] Ludwig M. Leng G. (2006). Dendritic peptide release and peptide-dependent behaviours. Nat. Rev. Neurosci. 7, 126–136 10.1038/nrn1845 16429122

[B34] Marsh A. A. Finger E. C. Mitchell D. G. V. Reid M. E. Sims C. Kosson D. S. (2008). Reduced amygdala response to fearful expressions in children and adolescents with callous-unemotional traits and disruptive behavior disorders. Am. J. Psychiatry 165, 712–720 10.1176/appi.ajp.2007.07071145 18281412

[B35] Nelder J. Wedderburn R. (1972). Generalized linear models. J. R. Statist. Soc. A 135, 370–384

[B36] Nitschke J. B. Nelson E. E. Rusch B. D. Fox A. S. Oakes T. R. Davidson R. J. (2004). Orbitofrontal cortex tracks positive mood in mothers viewing pictures of their newborn infants. Neuroimage 21, 583–592 10.1016/j.neuroimage.2003.10.005 14980560

[B37] Numan M. Insel T. R. (2003). Neurobiology of Parental Behavior. New York, NY: Springer

[B38] Numan M. Woodside B. (2010). Maternity: neural mechanisms, motivational processes, and physiological adaptations. Behav. Neurosci. 124, 715–741 10.1037/a0021548 21133530

[B39] Olazabal D. E. Young L. J. (2006). Oxytocin receptors in the nucleus accumbens facilitate “spontaneous” maternal behavior in adult female prairie voles. Neuroscience 141, 559–568 10.1016/j.neuroscience.2006.04.017 16725274

[B41] Parsons C. E. Stark E. A. Young K. S. Stein A. Kringelbach M. L. (2013). Understanding the human parental brain: a critical role of the orbitofrontal cortex. Soc. Neurosci. 8, 525–543 10.1080/17470919.2013.842610 24171901

[B43] Ragnauth A. K. Devidze N. Moy V. Finley K. Goodwillie A. Kow L. M. (2005). Female oxytocin gene-knockout mice, in a semi-natural environment, display exaggerated aggressive behavior. Gene. Brain Behav. 4, 229–239 10.1111/j.1601-183X.2005.00118.x 15924555

[B44] Riem M. M. E. Bakermans-Kranenburg M. J. Pieper S. Tops M. Boksem M. A. S. Vermeiren R. R. J. M. (2011a). Oxytocin modulates amygdala, insula, and inferior frontal gyrus responses to infant crying: a randomized controlled trial. Biol. Psychiatry 70, 291–297 10.1016/j.biopsych.2011.02.006 21470595

[B45] Riem M. M. E. Pieper S. Out D. Bakermans-Kranenburg M. J. van Ijzendoorn M. H. (2011b). Oxytocin receptor gene and depressive symptoms associated with physiological reactivity to infant crying. Soc. Cogn. Affect. Neurosci. 6, 294–300 10.1093/scan/nsq035 20400491 PMC3110427

[B46] Robinson E. A. Eyberg S. M. (1981). The dyadic parent-child interaction coding system: standardization and validation. J. Consult. Clin. Psychol. 49, 245–250 10.1037/0022-006X.49.2.245 7217491

[B47] Rodrigues S. M. Saslow L. R. Garcia N. John O. P. Keltner D. (2009). Oxytocin receptor genetic variation relates to empathy and stress reactivity in humans. Proc. Natl. Acad. Sci. U.S.A. 106, 21437–21441 10.1073/pnas.0909579106 19934046 PMC2795557

[B48] Rolls E. T. (2000). The orbitofrontal cortex and reward. Cereb. Cortex 10, 284–294 10.1093/cercor/10.3.284 10731223

[B49] Ross H. E. Cole C. D. Smith Y. Neumann I. D. Landgraf R. Murphy A. Z. (2009). Characterization of the oxytocin system regulating affiliative behavior in female prairie voles. Neuroscience 162, 892–903 10.1016/j.neuroscience.2009.05.055 19482070 PMC2744157

[B50] Ross H. E. Young L. J. (2009). Oxytocin and the neural mechanisms regulating social cognition and affiliative behavior. Front. Neuroendocrinol. 30, 534–547 10.1016/j.yfrne.2009.05.004 19481567 PMC2748133

[B51] Saphire-Bernstein S. Way B. M. Kim H. S. Sherman D. K. Taylor S. E. (2011). Oxytocin receptor gene (OXTR) is related to psychological resources. Proc. Natl. Acad. Sci. U.S.A. 108, 15118–15122 10.1073/pnas.1113137108 21896752 PMC3174632

[B52] Stoop R. (2012). Neuromodulation by oxytocin and vasopressin. Neuron 76, 142–159 10.1016/j.neuron.2012.09.025 23040812

[B53] Swain J. E. (2011). The human parental brain: *in vivo* neuroimaging. Prog. Neuropsychopharmacol. Biol. Psychiatry 35, 1242–1254 10.1016/j.pnpbp.2010.10.017 21036196 PMC4329016

[B54] Swain J. E. Leckman J. F. Mayes L. C. Feldman R. Constable R. T. Schultz R. T. (2004). Neural substrates and psychology of human parent–infant attachment in the postpartum. Biol. Psychiatry 55, 153S Available online at: http://ac.els-cdn.com.proxy-um.researchport.umd.edu/S0006322304002446/1-s2.0-S0006322304002446-main.pdf?_tid=b496afc0-82e6-11e3-bc85-00000aab0f01&acdnat=1390341493_c129ae5a265d822778872839cb6d4bfe

[B55] Swain J. E. Lorberbaum J. P. Kose S. Strathearn L. (2007). Brain basis of early parent-infant interactions: psychology, physiology, and in vivo functional neuroimaging studies. J. Child Psychol. Psychiatry 48, 262–287 10.1111/j.1469-7610.2007.01731.x 17355399 PMC4318551

[B56] Swain J. E. Tasgin E. Mayes L. C. Feldman R. Constable R. T. Leckman J. F. (2008). Maternal brain response to own baby cry is affected by cesarean section delivery. J. Child Psychol. Psychiatry 49, 1042–1052 10.1111/j.1469-7610.2008.01963.x 18771508 PMC3246837

[B57] Takayanagi Y. Yoshida M. Bielsky I. F. Ross H. E. Kawamata M. Onaka T. (2005). Pervasive social deficits, but normal parturition, in oxytocin receptor-deficient mice. Proc. Natl. Acad. Sci. U.S.A. 102, 16096–16101 10.1073/pnas.0505312102 16249339 PMC1276060

[B58] Tost H. Kolachana B. Hakimi S. Lemaitre H. Verchinski B. A. Mattay V. S. (2010). A common allele in the oxytocin receptor gene (OXTR) impacts prosocial temperament and human hypothalamic-limbic structure and function. Proc. Natl. Acad. Sci. U.S.A. 107, 13936–13941 10.1073/pnas.1003296107 20647384 PMC2922278

[B59] van den Burg E. H. Neumann I. D. (2011). Bridging the gap between GPCR activation and behaviour: Oxytocin and prolactin signalling in the hypothalamus. J. Mol. Neurosci. 43, 200–208 10.1007/s12031-010-9452-8 20865346

[B60] Volkow N. D. Tomasi D. Wang G. J. Fowler J. S. Telang F. Goldstein R. Z. (2011). Positive emotionality is associated with baseline metabolism in orbitofrontal cortex and in regions of the default network. Mol. Psychiatry 16, 818–825 10.1038/mp.2011.30 21483434 PMC3137758

[B60a] Webster-Stratton C. (1998). Preventing conduct problems in head start children: strengthening parenting competencies. J. Consult. Clin. Psychol. 66, 715–730 9803690 10.1037//0022-006x.66.5.715

[B61] Wegman H. L. Stetler C. (2009). A meta-analytic review of the effects of childhood abuse on medical outcomes in adulthood. Psychosom. Med. 71, 805–812 10.1097/PSY.0b013e3181bb2b46 19779142

[B62] Worsley K. J. Marrett S. Neelin P. Vandal A. C. Friston K. J. Evans A. C. (1996). A unified statistical approach for determining significant signals in images of cerebral activation. Hum. Brain Mapp. 4, 58–73 10.1002/(SICI)1097-0193(1996)4:1<58::AID-HBM4>3.0.CO;2-O 20408186

