# Supplementary material for: Genetic imaging of the association of oxytocin receptor gene (OXTR) polymorphisms with positive maternal parenting
Source: Front Behav Neurosci. 2014 Jan 3;8:21. doi: 10.3389/fnbeh.2014.00021 (PMC3909919; doi:10.3389/fnbeh.2014.00021)
Supplement: Supplemental Figure S1 — Flowchart of the sequenced statistical analyses. [file DataSheet1.DOCX]

SUPPLEMENTAL MATERIALS

Genetic Imaging of the Association of Oxytocin Receptor Gene (OXTR) Polymorphisms with Positive Maternal Parenting

Kalina J. Michalska, Jean Decety, Chunyu Liu, Qi Chen, Meghan Overmyer Martz, Suma Jacob, Alison E. Hipwell, Steve S. Lee, Andrea Chronis-Tuscano, Irwin D. Waldman, &

Benjamin B. Lahey

Supplemental Table S1. Demographic and birth characteristics of three subgroups of a total 40 mothers and their children participating in tests of associations between different variables.

Scanned and

Scanned Genotyped Genotyped

Participants Participants Participants

N = 34* N = 35* N = 30*

Mother

Age at scan (mean, SD) 47.41 (5.23) 47.51 (5.07) 47.50 (5.32)

Number of live births by scan (mean, SD) 2.35 (0.73) 2.37 (0.69) 2.40 (0.74)

Child

Child’s age in wave 1 in years (mean, SD) 5.24 (0.78) 5.26 (0.78) 5.20 (0.80)

Sex (% male) 85.29 85.71 86.67

Race-ethnicity (% African American) 47.06 42.86 43.33

Birth order (% first born) 55.88 51.43 50.00

Prematurity in weeks (mean, SD) 0.38 (0.92) 0.37 (0.91) 0.43 (0.97)

Delivery (% Caesarian) 26.47 22.86 26.678

ADHD diagnosis in wave 1 (% present) 50.00 51.43 50.00

Child disruptive behavior in wave (z) -0.13 (0.62) -0.08 (0.61) -0.074 (0.64)

_____________________

*After removing one participant with missing parenting data.

Supplemental Table S2

Areas of significant hemodynamic activation when viewing a picture of own child > unrelated child.

|  |  | MNI Coordinates | | |  |
| --- | --- | --- | --- | --- | --- |
| Brain Region | Side | X | Y | z | t-value |
| **Activated Regions** |  |  |  |  |  |
| Medial prefrontal cortex | R | 4 | 50 | 22 | 3.24 |
| Medial prefrontal cortex | L | -4 | 56 | -4 | 2.53 |
| Orbitofrontal cortex | R | 2 | 54 | -4 | 3.40* |
| Orbitofrontal cortex | L | -2 | 54 | -4 | 3.03 |
| Dorsolateral prefrontal cortex | R | 22 | 38 | 48 | 2.67 |
| Dorsolateral prefrontal cortex | L | -18 | 40 | 46 | 2.73 |
| Superior frontal gyrus | L | -20 | 16 | 50 | 2.53 |
| Middle frontal gyrus | R | 40 | 12 | 32 | 2.62 |
| Middle frontal gyrus | L | -30 | 14 | 44 | 3.27 |
| Inferior frontal gyrus | R | 48 | 12 | 14 | 3.49* |
| Inferior frontal gyrus | L | -58 | 4 | 28 | 2.84 |
| Anterior cingulate cortex | R | 10 | 52 | 12 | 2.14 |
| Anterior cingulate cortex | L | -10 | 44 | 10 | 3.33 |
| Anterior insula | L | -32 | 14 | -2 | 3.78* |
| Fusiform gyrus | R | 36 | -42 | -22 | 4.14* |
| Fusiform gyrus | L | -32 | -36 | -24 | 4.09* |
| Superior parietal lobe | R | 28 | -58 | 48 | 2.48 |
| Superior parietal lobe | L | -30 | -56 | 50 | 3.15 |
| Inferior parietal lobe | L | -40 | -54 | 46 | 2.68 |
| Posterior cingulate cortex | R | 12 | -56 | 6 | 4.57* |
| Posterior cingulate cortex | L | -12 | -60 | 14 | 5.07* |
| Precuneus | R | 18 | -58 | 24 | 4.69* |
| Precuneus | L | -16 | -58 | 16 | 5.77* |
| Thalamus | R | 16 | -22 | 10 | 3.22 |
| Thalamus | L | -6 | -24 | 12 | 2.35 |
| Amygdala | R | 32 | 0 | -22 | 2.06 |
| Putamen | L | -16 | 2 | 4 | 2.37 |
| Midbrain (brainstem) | R | 16 | -20 | -18 | 4.64* |
| Midbrain (brainstem) | L | 0 | -18 | -20 | 4.11* |
| Periaqueductal gray | R | 4 | -30 | -14 | 3.33* |
| Periaqueductal gray | L | -4 | -32 | -12 | 2.91 |
| Cerebellum- anterior lobe | R | 6 | -58 | -28 | 3.76* |
| Cerebellum- anterior lobe | L | -6 | -54 | -24 | 3.70* |
| Cerebellum- posterior lobe | R | 18 | -68 | -28 | 4.44* |
| Cerebellum- posterior lobe | L | -10 | -76 | -24 | 4.07* |

Activations in regions without an asterisk = P < 0.005, uncorrected; * P < 0.05 family-wise error (voxel-wise) corrected using small-volume correction.

Supplemental Table S3

Areas of significant activation and deactivation when viewing a person behaving inappropriately > behaving neutrally in the own child condition.

|  |  | MNI Coordinates | | |  |
| --- | --- | --- | --- | --- | --- |
| Brain Region | Side | x | Y | z | t-value |
| **Activated Regions** |  |  |  |  |  |
| Medial prefrontal cortex | R | 2 | 54 | 26 | 5.20* |
| Dorsolateral prefrontal cortex | R | 26 | 56 | 28 | 5.02* |
| Dorsolateral prefrontal cortex | L | -24 | 52 | 28 | 4.82* |
| Inferior frontal gyrus | L | -54 | 12 | 22 | 3.99* |
| Anterior cingulate cortex | R | 4 | 50 | 24 | 4.18* |
| Anterior cingulate cortex | L | -2 | 36 | 22 | 3.11 |
| Mid-Insula | R | 38 | -12 | -4 | 2.09 |
| Mid-Insula | L | -42 | 8 | -2 | 3.78* |
| Fusiform gyrus | R | 48 | -40 | -26 | 3.43* |
| Fusiform gyrus | L | -44 | -44 | -22 | 2.62 |
| Posterior superior temporal sulcus | R | 58 | -36 | 22 | 3.39* |
| Posterior superior temporal sulcus | L | -52 | -34 | 28 | 7.80* |
| Temporal pole | R | 34 | 20 | -28 | 4.57* |
| Temporal pole | L | -30 | 6 | -26 | 2.74 |
| Inferior parietal lobe | R | 64 | -40 | 26 | 4.85* |
| Inferior parietal lobe | L | -56 | -38 | 52 | 4.74* |
| Posterior cingulate cortex | R | 10 | -42 | 24 | 4.39* |
| Posterior cingulate cortex | L | -4 | -50 | 28 | 5.03* |
| Precuneus | R | 18 | 64 | 30 | 3.33 |
| Precuneus | L | -10 | -64 | 36 | 4.96* |
| Thalamus | R | 18 | -28 | 6 | 3.45* |
| Thalamus | L | -20 | -18 | 6 | 4.52* |
| Amygdala | L | -18 | 0 | -18 | 3.38* |
| Hippocampus | R | 24 | -32 | -2 | 3.37* |
| Hippocampus | L | -14 | -28 | -12 | 5.75* |
| Caudate | R | 14 | -12 | 22 | 3.45* |
| Caudate | L | -14 | -12 | 22 | 2.78 |
| Putamen | R | 28 | 0 | 0 | 3.01 |
| Putamen | L | -32 | 2 | -2 | 3.36* |
| Midbrain | R | 2 | -28 | -10 | 4.28* |
| Midbrain | L | -2 | -28 | -10 | 4.02* |
| Periaqueductal gray | R | 2 | -24 | -26 | 3.92* |
| Periaqueductal gray | L | -2 | -24 | -24 | 4.5* |
| Cerebellum- anterior lobe | R | 36 | -48 | -36 | 6.02* |
| Cerebellum- anterior lobe | L | -30 | -58 | -34 | 5.32* |
| Cerebellum- posterior lobe | R | 24 | -76 | -28 | 6.54* |
| Cerebellum- posterior lobe | L | -12 | -74 | -16 | 5.11* |
| **Deactivated Regions** |  |  |  |  |  |
| Somatosensory cortex | R | 44 | -24 | 42 | 4.21* |
| Somatosensory cortex | L | -46 | -24 | 52 | 5.18* |
| Posterior insula | R | 40 | -10 | 10 | 4.30* |
| Posterior insula | L | -36 | -18 | 10 | 4.94* |

Activations in regions without an asterisk = P < 0.005, uncorrected; * P < 0.05 family-wise error (voxel-wise) corrected using small-volume correction.

Supplemental Table S4. Allele frequencies of OXTR SNPs by ancestry groups.

European African

American American Fisher’s Exact Test

n = 21 n = 15 P =

rs53576

A 14 9 0.8031

G 28 21

rs1042778

T 13 19 0.0085

G 29 11

Note: Using PEDSTATS 0.6.12 (Wiggington & Abecasis, 2005),^66^ the alleles of each of polymorphism were in Hardy-Weinberg equilibrium (*OXTR*  rs1042778 p = 0.519, *OXTR*  rs53576 p = 0.439).


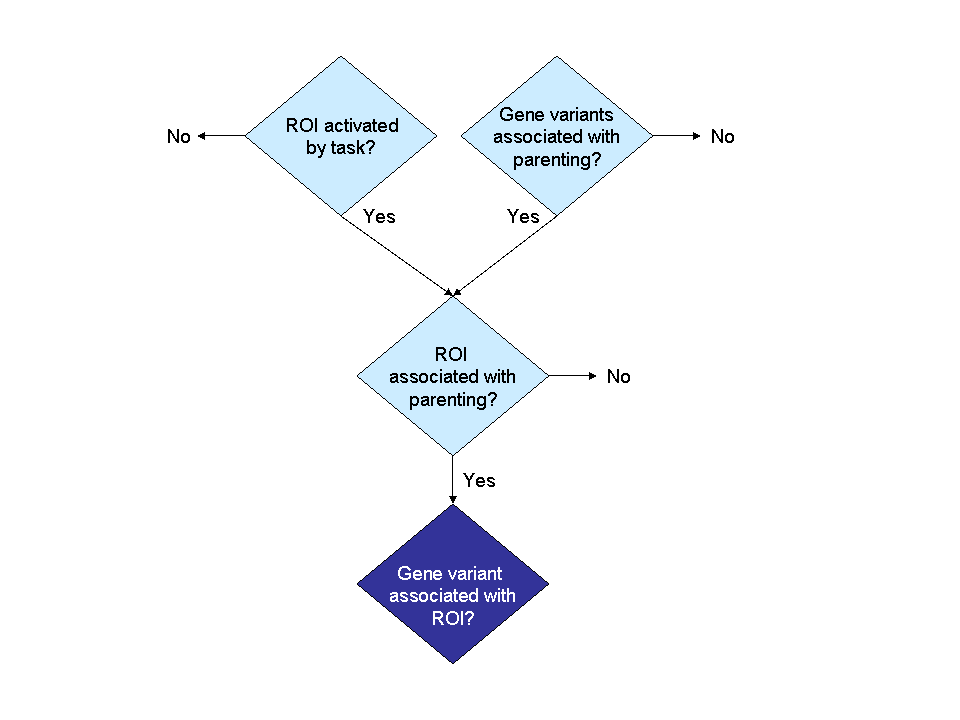


Supplemental Figure S1. Flowchart of the sequenced statistical analyses.
